# Pupylated proteins are subject to broad proteasomal degradation specificity and differential depupylation

**DOI:** 10.1371/journal.pone.0215439

**Published:** 2019-04-22

**Authors:** Juerg Laederach, Hengjun Cui, Eilika Weber-Ban

**Affiliations:** Institute of Molecular Biology and Biophysics, Department of Biology, ETHZ, Zurich, Switzerland; University of British Columbia, CANADA

## Abstract

In actinobacteria, post-translational modification of proteins with prokaryotic ubiquitin-like protein Pup targets them for degradation by a bacterial proteasome assembly consisting of the 20S core particle (CP) and the mycobacterial proteasomal ATPase (Mpa). Modification of hundreds of cellular proteins with Pup at specific surface lysines is carried out by a single Pup-ligase (PafA, proteasome accessory factor A). Pupylated substrates are recruited to the degradative pathway by binding of Pup to the N-terminal coiled-coil domains of Mpa. Alternatively, pupylation can be reversed by the enzyme Dop (deamidase of Pup). Although pupylated substrates outcompete free Pup in proteasomal degradation, potential discrimination of the degradation complex between the various pupylated substrates has not been investigated. Here we show that Mpa binds stably to an open-gate variant of the proteasome (oCP) and associates with bona fide substrates with highly similar affinities. The proteasomal degradation of substrates differing in size, structure and assembly state was recorded in real-time, showing that the pupylated substrates are processed by the Mpa-oCP complex with comparable kinetic parameters. Furthermore, the members of a complex, pupylated proteome (pupylome) purified from *Mycobacterium smegmatis* are degraded evenly as followed by western blotting. In contrast, analysis of the depupylation behavior of several pupylome members suggests substrate-specific differences in enzymatic turnover, leading to the conclusion that largely indiscriminate degradation competes with differentiated depupylation to control the ultimate fate of pupylated substrates.

## Introduction

In mycobacteria and many other actinobacteria, covalent modification of proteins with the small (60–70 residues), intrinsically disordered protein Pup (prokaryotic ubiquitin-like protein) allows them to be recognized and degraded by a bacterial proteasome complex consisting of the 20S proteasome core particle and a ring-shaped ATPase referred to as Mpa (mycobacterial proteasomal ATPase) in mycobacteria or ARC (ATPase forming ring-shaped complexes) in other actinobacteria [1, 2]. Although this proteasomal degradation pathway is not essential under standard culture conditions, it provides bacteria with a critical advantage under certain stress conditions [3]. For example, the human pathogen *Mycobacterium tuberculosis* (Mtb) makes use of this pathway to persist inside host macrophages, while its non-pathogenic relative *Mycobacterium smegmatis* (Msm) gains advantage from the Pup-proteasome system under nitrogen starvation and DNA damage stress [4–8]. The modification of cellular proteins with Pup involves two structurally homologous and evolutionarily related enzymes, the Pup ligase PafA (proteasome accessory factor A) and the Pup deamidase/depupylase Dop (deamidase of Pup) [9–11]. The Pup ligase attaches the side-chain carboxylate of the C-terminal glutamate residue of Pup to a lysine side chain in the target protein by forming an isopeptide bond [12–15]. In mycobacteria, Pup is encoded with a C-terminal glutamine that first must be deamidated to glutamate by the enzyme Dop to produce the side-chain carboxylate for ligation [10]. The cleavage of the C-N bond in the glutamine side chain is chemically equivalent to the cleavage of the isopeptide bond of a pupylated protein. Hence it is not surprising that Dop also catalyzes the depupylation reaction [16–18]. Recognition of pupylated substrates by the proteasome complex occurs at the hexameric AAA+ Mpa-ring [19, 20], which is formed of three parts: one ring tier is made of the C-terminal AAA+ modules that stack on top of the 20S particle, followed N-terminally by a narrower collar-like double-ring tier formed by two consecutive β-barrel domains [21, 22]. From this tier emerge N-terminal helices that are pairwise engaged into a total of three coiled-coils. Upon binding to Mpa, Pup adopts a helical conformation in part of its sequence and associates via this helix with the coiled-coils of Mpa, forming a shared, three stranded coil [23, 24]. Pup’s N-terminus remains unstructured and available for threading into the Mpa central pore. Pup binds to Mpa with a 1:1 stoichiometry despite the presence of three coiled-coils [14], which is due to space constraints in the vicinity of the Mpa ring pore [20]. Mpa, similar to eukaryotic and archaeal proteasomal ATPases, uses a C-terminal interaction motif with a penultimate tyrosine to dock onto the 20S core particle. However, efficient degradation of pupylated substrates by the Mpa-proteasome *in vitro* could only be observed when an open-gate variant of the 20S core particle (oCP) is used, in which the N-termini of the α-subunits are truncated by seven residues [21, 25]. A recent X-ray structure of Mpa suggests that the reason might be found in the formation of a stable β-grasp domain at the very C-terminus of Mpa burying the C-terminal GQYL interaction motif inside the Mpa central pore [22]. It was suggested that association even with the oCP is strongly hindered by the formation of this domain. This, however, contradicts earlier studies that have shown stable interaction between Mpa and oCP *in vitro* using size exclusion chromatography or electron microscopy [20, 26].

The pupylated proteome modified on one or more lysine side chains with Pup, referred to as the pupylome, was shown in different organisms to encompass several hundred proteins of varying size, structure and assembly state [8, 27–34]. Nevertheless, steady-state levels of known pupylation substrates only accumulate for a subset of pupylome members in proteasome-deficient mutant strains of mycobacteria, suggesting that pupylation does not necessarily lead to degradation. The regulation contributing to the fate of a pupylated protein could occur at multiple levels, including the interplay of pupylation and depupylation, but also the recruitment and degradation of pupylated substrates by the proteasome complex itself [5, 26, 35, 36]. Using a combination of NMR experiments and biochemical analysis we have recently shown that the intrinsically disordered Pup remains unstructured when ligated to two well-established pupylation substrates, retaining the ability to interact with its different interaction partners and ruling out the conformation of Pup on the substrate as a decisive element for substrate fate [37].

It was shown previously that pupylated substrates outcompete free Pup *in vitro* for recruitment to the proteasome complex, suggesting interactions between the substrate portion and the proteasome in addition to Pup binding [25]. It is therefore conceivable that the proteasome complex displays some specificity in the selection of substrates or in the way they are processed by the ATPase. In this study we investigate the recruiting ATPase-ring Mpa of the proteasome complex as a potential source for substrate discrimination. We analyze the substrate binding and processing parameters of several well-established pupylation substrates *in vitro* and, using affinity purified pupylome from *Mycobacterium smegmatis* (Msm), carry out *in vitro* degradation on the entire pupylated proteome. Our results show that the Mpa-proteasome complex is remarkably indiscriminate in its degradation of pupylated proteins of diverse size, stability and assembly state, suggesting that all pupylated substrates are invariably turned over by the proteasome when given access. *In vitro* depupylation by Dop of several pupylome members shows substrate-specific differences in enzymatic turnover, leading to the conclusion that competition of largely indiscriminate degradation with differentiated depupylation or regulation of pupylation itself, determine the fate of pupylome members.

## Results

### The mycobacterial proteasomal ATPase ring binds to the open-gate 20S particle with low micromolar affinity

**Fig 1:**
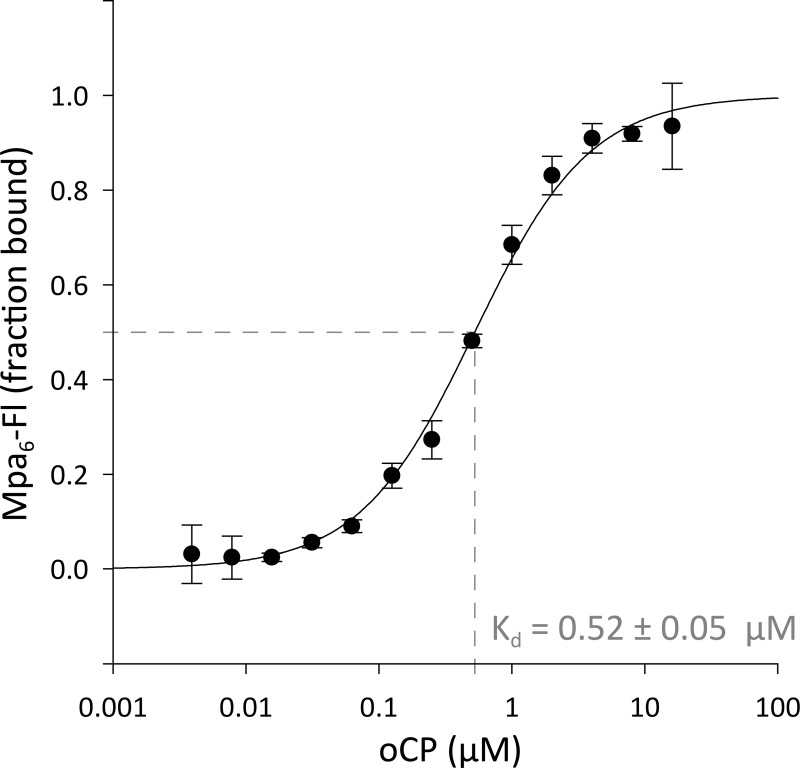
Binding of wild-type Mpa to open-gate proteasome. Open-gate ^Mtb^proteasome (oCP) at concentrations from 16 μM to 4 nM was titrated against 5 nM fluorescein-labelled ^Mtb^Mpa and their association probed with Microscale Thermophoresis (MST) in three replicates. The dependence of the bound fraction of Mpa (from normalized fluorescence signal change) on the proteasome concentration was fitted with the company-provided analysis software, yielding a dissociation constant of 0.52 ± 0.05 μM.

As a recent X-ray structure of Mpa revealed the presence of a stable β-grasp domain at the very C-terminus of Mpa burying the C-terminal interaction motif of Mpa inside the Mpa central pore, it was suggested by the authors that association even with the open gate proteasome is significantly impaired by the formation of this domain [22]. Therefore, to quantitatively assess the ability of Mpa to form a stable complex with oCP *in vitro*, we used microscale thermophoresis (MST) to determine the dissociation constant between Mpa and oCP. Fluorescently labeled Mpa (Mpa-Fl) was titrated with oCP and subjected to a temperature jump from 17°C to 24°C, resulting in differential thermophoretic movement of the Mpa-oCP complex versus Mpa alone (Fig 1) [38]. Using the company-provided analysis tool to fit the recorded thermophoretic data, a K_d_ of 0.52 ± 0.05 μM was determined for binding of the Mpa-ring to the oCP, indicating that Mpa interacts stably with oCP at the concentrations used in the *in vitro* assays performed in this study.

### In absence of ATP-dependent pore engagement, Mpa binds pupylated substrates uniformly with at least an order of magnitude higher affinity than Pup alone

**Fig 2:**
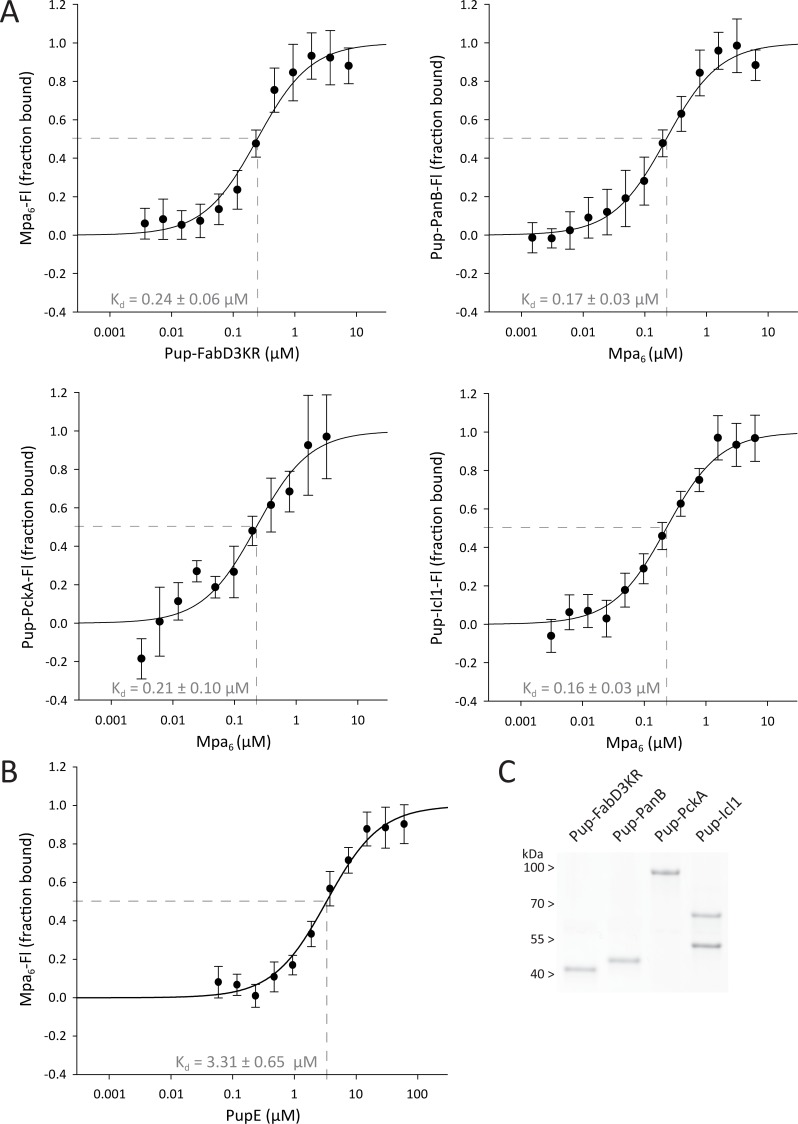
Association of four different pupylated substrates or free Pup to Mpa probed by microscale thermophoresis. (A) Binding of Pup-substrate to wild type Mpa in the absence of nucleotide. Data was acquired with MST in 4–8 replicates, by titrating proteins from 7 μM to 3 nM against their labeled binding partner. For pupylated PanB, PckA and Icl1, fixed concentrations of Pup-substrate-Fl were used (70, 40 and 150 nM protomer) and Mpa was titrated to the reaction, while for Pup-FabD3KR, the substrate was titrated against 5 nM of labeled Mpa hexamer for a better signal to noise ratio. The dependence of the bound fraction of fluorescein-labeled molecule (from normalized fluorescence signal change) on the concentrations of binding partner was fitted with the company-provided analysis software, yielding dissociation constants of 0.24 ± 0.06 μM for Pup-FabD3KR, 0.17 ± 0.03 μM for Pup-PanB, 0.21 ± 0.10 μM for Pup-PckA and 0.16 ± 0.03 μM for Pup-Icl1. (B) Binding of PupE to wild type Mpa in the absence of nucleotide. Data was acquired with MST in 5 replicates, by titrating Pup from 60 μM to 40 nM against 5 nM fluorescently labeled Mpa hexamer. Fitting of the thermophoresis binding curve with the company-provided analysis software yielded a dissociation constant of 3.31 ± 0.65 μM. (C) Purified pupylated substrates analyzed on Coomassie stained SDS-PA gel. The target proteins used in this study were heterologously expressed in *E*. *coli*, purified and subsequently pupylated *in vitro* at 3 μM concentration with 5 molar equivalents His_10_-3C-^Mtb^PupE and 1/3 molar equivalents ^Mtb^PafA, before immobilized metal affinity chromatography (IMAC) to ensure clean and homogeneous sample. While FabD3KR, PanB and PckA could be pupylated at every protomer, the homotetrameric Icl1 had only two out of four subunits pupylated.

Next, we investigated the interaction of pupylated degradation substrates with Mpa. MST was used to measure binding of the known genuine Mtb pupylation substrates malonyl transacylase FabD, ketopantoate hydroxymethyltransferase PanB, phosphoenolpyruvate carboxykinase PckA and isocitrate lyase 1 Icl1 to Mpa. To obtain homogeneously pupylated FabD, a triple lysine-to-arginine variant (FabD3KR) was used, removing three secondary pupylation sites and leaving only the main site intact [37]. As the experiments were performed under the exclusion of nucleotide, only coiled-coil interactions between Pup and Mpa, as well as potential unspecific interactions between substrate and Mpa, but not active pore engagement contribute to the binding. While for pupylated PanB, Icl1 and PckA, Mpa hexamer could be titrated to a constant concentration of fluorescein-labeled Pup-substrate, the experimental setup had to be reversed for Pup-FabD3KR to obtain sufficient signal (i.e. fluorescein-labeled Mpa at constant concentration was titrated with Pup-FabD3KR) (Fig 2A). The dissociation constants determined for the different substrates are within error identical with an average of 0.20 ± 0.06 μM. Pup on its own displayed a one order of magnitude higher K_d_ of 3.31 μM as determined by MST in this study (Fig 2B), which is in agreement with previous data obtained using anisotropy and isothermal titration calorimetry measurements [19]. This suggests that not only the covalently attached Pup moiety, but also the substrate portion itself contributes to the binding to Mpa [19].

### Proteasomal degradation of diverse pupylated substrates exhibits a narrow range of kinetic parameters

**Fig 3:**
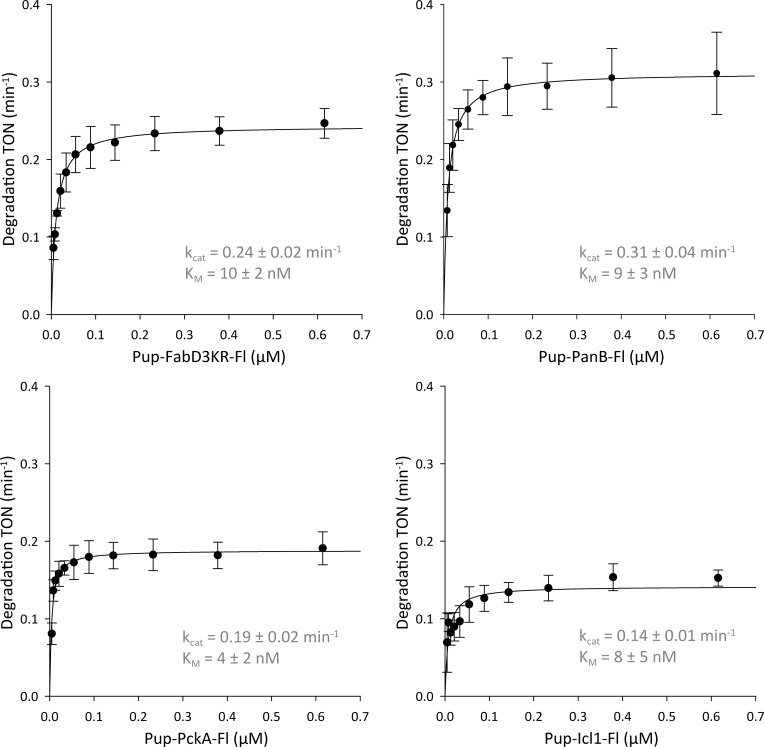
Michalis-Menten analysis for the degradation of Pup-substrate by the Mpa-oCP complex. Turnover numbers (min^-1^ per Mpa-oCP complex) were calculated from the initial phase (within ≤ 10% signal change) of fluorescein-labeled Pup-substrate degradation at excess substrate concentrations. With the exception of Pup-PanB-Fl, pupylated substrates were used in a ratio of pupylated substrate protomer to Mpa of 1:10. Pup-PanB-Fl was used in a ratio of substrate protomer to Mpa of 1:100. The monomeric FabD3KR and PckA and the decameric PanB were thus in 10-fold excess over Mpa hexamer, the tetrameric Icl1 in 5-fold excess. Degradation curves were measured exciting at λ_ex_ = 485 nm and recording the emission at λ_em_ = 525 nm in a plate reader. TONs were plotted with a confidence interval of 2x standard deviation against the corresponding concentrations of Pup-substrate-Fl with subsequent fitting to the Michaelis-Menten equation: TON = (k_cat_ * [S]) / (K_M_ + [S]). The k_cat_ values for the reactions range between 0.14 and 0.31 min^-1^, while the K_M_ values fall into a narrow, low nanomolar range (4–10 nM) (Table 1).

Having demonstrated that the affinity of pupylated substrates to the proteasomal ATPase does not differ significantly between substrates, next we wanted to assess whether processing by the Mpa-proteasome complex in presence of nucleotide would exhibit substrate-dependent differences. To this end we followed the degradation of fluorescein-labeled, pupylated substrates by the Mpa-oCP complex *in vitro*. Breakdown of the protein leads to unquenching of the fluorophore and the degradation time course can be followed by measuring the resulting increase in fluorescence. From the dependence of the initial substrate degradation rates on substrate concentration, the catalytic parameters (k_cat_ and K_M_) were determined for the degradation reactions of the various substrates employing a 10-fold excess of pupylated substrate protomer over Mpa ring, with the exception of Pup-PanB-Fl where a 100-fold excess was used (Fig 3). Consequently, in terms of substrate molecule rather than Pup-ligated protomer, the monomeric FabD3KR and PckA and the decameric PanB were in 10-fold excess over Mpa hexamer, the tetrameric Icl1 in 5-fold excess. Of the substrates used for this study, FabD and PckA, though both monomeric, have significantly different molecular weights of 31 and 67 kDa, respectively. PanB is a 293 kDa, homodecameric complex with all subunits singly pupylated *in vitro* and Icl1 is a homotetramer with 188 kDa complex size, where only two out of four subunits are pupylated *in vitro* (Fig 2C). Considering the diverse nature of these substrates, the observed k_cat_ and K_M_ parameters fall into a narrow range (Table 1). The maximal substrate turnover for all measured substrates lies between 0.14 to 0.31 min^-1^ and the Michaelis constants also fall into a narrow range exhibiting values between 4 and 10 nM. That the observed Michaelis constants are significantly lower than the measured K_d_ values for the same substrates likely is due to the ATPase-driven irreversible nature of the translocation through the Mpa pore.

**Table 1:** Kinetic parameters for degradation of four different Pup-substrates by the Mpa-oCP complex.

	k_cat_ (min^-1^)	K_M_ (nM)	k_cat_/K_M_ (M^-1^ s^-1^)
Pup-FabD3KR-Fl	0.24 ± 0.02	10 ± 2	3.9 x 10^5^
Pup-PanB-Fl	0.31 ± 0.04	9 ± 3	5.7 x 10^5^
Pup-PckA-Fl	0.19 ± 0.02	4 ± 2	7.4 x 10^5^
Pup-Icl1-Fl	0.14 ± 0.01	8 ± 5	3.1 x 10^5^

Both k_cat_ and K_M_ for the degradation of the different substrates fall into a narrow range. The Mpa-oCP complex can degrade pupylated substrate at rates between 0.14 and 0.31 min^-1^, depending on the respective protein. K_M_ values for the reactions lie in the low nanomolar range, suggesting that once engaged by the Mpa pore, the Pup-substrates are translocated into the 20S core and degraded.

### Affinity-purified pupylated proteome is degraded evenly by the Mpa-proteasome *in vitro*

**Fig 4:**
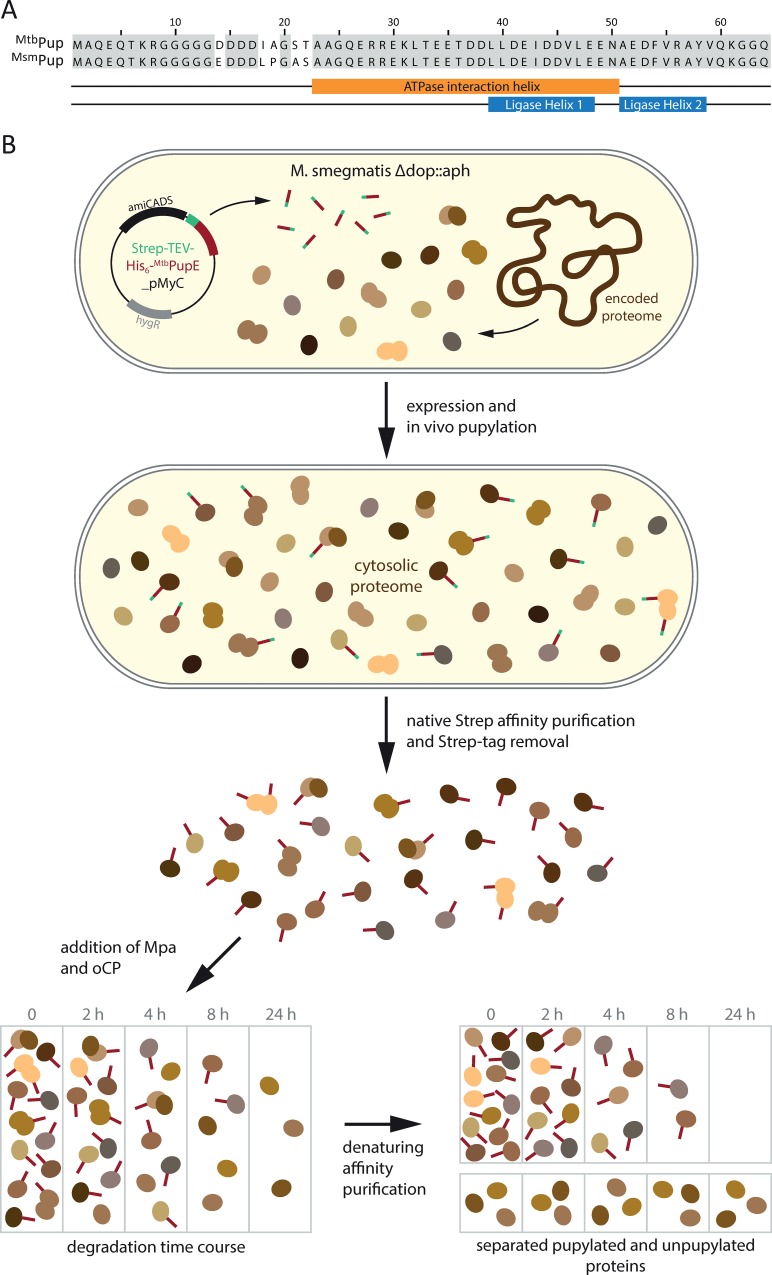
Pupylome production and degradation. (A) Mtb and Msm Pup share high sequence identity. While a few residues differ between ^Mtb^Pup used for all experiments in this study and ^Msm^Pup, both the ATPase and ligase interaction segments, as well as the N-terminal sequence serving as threading element for engaging the Mpa pore are identical. (B) Flow-scheme for pupylome production and degradation. Endogenous PafA in *M*. *smegmatis Δdop* (Msm*Δdop*) cells was used to modify substrates *in vivo* with Strep-TEV-His_6_-^Mtb^PupE, overexpressed from a plasmid. Pupylated substrates were purified via the Strep-tag on Pup. After proteolytic cleavage of the purification tag, purified wild-type Mpa and oCP were added to the reaction and samples were taken at the indicated time-points. Pupylated proteins remaining in the mixture were separated from secondary binders and the Mpa-oCP degradation complex via the His_6_-tag on Pup under denaturing conditions in 6 M guanidinium chloride. After protein precipitation with TCA/NaDoC, the samples were dissolved in SDS loading buffer and analyzed with SDS-PAGE and Western-blotting (Fig 5).

**Fig 5:**
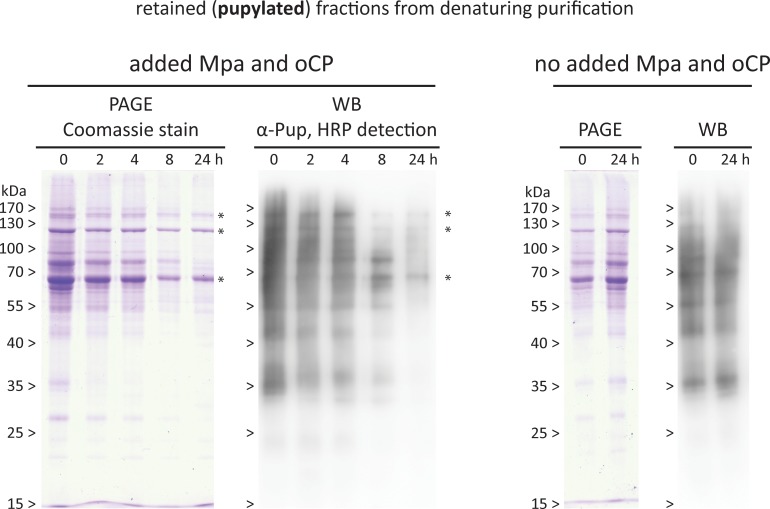
Degradation time-course of purified pupylome by the Mpa-oCP complex. Affinity-purified pupylome from Msm was subjected to degradation by the ^Mtb^Mpa-oCP complex along a 24 hour time course. Aliquots drawn along the time-course are visualized by Coomassie-stained SDS-PA gels (PAGE) and anti-Pup Western-blotting (WB). During the sampling time-course, almost all of the pupylated substrates from the mixture are degraded in an evenly distributed fashion (two left panels). Only three major bands stay visibly behind on the Coomassie-stained SDS-PA gel at roughly 150, 120 and below 70 kDa (indicated with a star), on Western blot three equivalent bands can be detected, although the two at 150 and 120 kDa are faint. The control without added ^Mtb^Mpa-oCP shows no loss of signal over the time of sampling.

The *in vitro* analysis of a subset of pupylated substrates suggests that the Mpa-proteasome complex does not discriminate between the different pupylated proteins. Nevertheless, it could be argued that despite the selection of substrate proteins with very distinct physical properties, we cannot exclude the possibility that some targets in the pupylome pool produced by mycobacteria exhibit unusual degradation behavior or could for steric reasons even be completely resistant to degradation, particularly since steady-state levels were found to remain unchanged upon proteasome-disruption for several pupylome members *in vivo* [30]. To investigate this possibility, we used affinity-purified whole pupylome as degradation pool for *in vitro* proteasomal degradation (Fig 4). A tandem Strep- and His_6_-tagged variant of ^Mtb^PupE was expressed in the model organism *M*. *smegmatis* mc^2^155 carrying a *dop* knockout mutation (Msm*Δdop*) [16], to ensure only the affinity-tagged Pup could be used to modify target proteins and no depupylation could take place (Fig 4B). We chose ^Mtb^PupE, as we had established all our *in vitro* assays with the Mtb Pup-proteasome system (PPS) proteins. Additionally, Mtb and Msm Pup are virtually identical with differences only in five of the 64 residues. Importantly, the sequence for Mpa and PafA binding as well as the N-terminus used for threading into the ATPase are identical (Fig 4A). Using Strep-affinity tag purification and subsequent removal of the Strep tag, we generated a natively folded, affinity-purified His_6_-Pup-tagged pupylome. Upon addition of Mpa and oCP, we took samples along a 24 hour degradation time course at 37°C, and subsequently purified them by metal-chelating affinity-purification under denaturing conditions to separate His_6_-Pup-tagged from untagged proteins (i.e. Mpa, proteasome and potential secondary/tertiary binders). The obtained samples were analyzed by SDS-PAGE and Western blotting (Fig 5).

The degradation time-course of the pupylated substrate fractions (Fig 5, left panels) shows an even disappearance of the protein bands across the entire molecular weight range, both in the Coomassie-stained gel as well as the anti-Pup Western blot, with the exception of three bands remaining visible on the Coomassie-stained SDS-PA gel (marked by stars). Equivalent bands are detected on the anti-Pup Western blot, however, the two at 150 kDa and 120 kDa appear much fainter. This could suggest that despite the denaturing purification, a portion of these two proteins may be unpupylated and might have co-purified with the pupylome. The control samples without added Mpa-oCP show no loss in signal over 24 hours (Fig 5, right panels), indicating that proteasomal degradation is solely responsible for the disappearance of the bands. The bands on the blot are less sharp than the bands on the Coomassie-stained gel, so that the higher molecular weight bands are less well separated and present as a smear. Furthermore, the Coomassie-stained bands are more intense for the large proteins due to the fact that more dye binds, while the intensity of the blot depends on the (in many cases single) Pup attached to the protein. We noted one band above the 25 kDa marker that appears strong in the Coomassie gel but shows no equivalent band on the blot. As this band disappears along with the general pupylome, we suspect that, for unknown reasons, the antibody does not stain this pupylated protein well.

**Fig 6:**
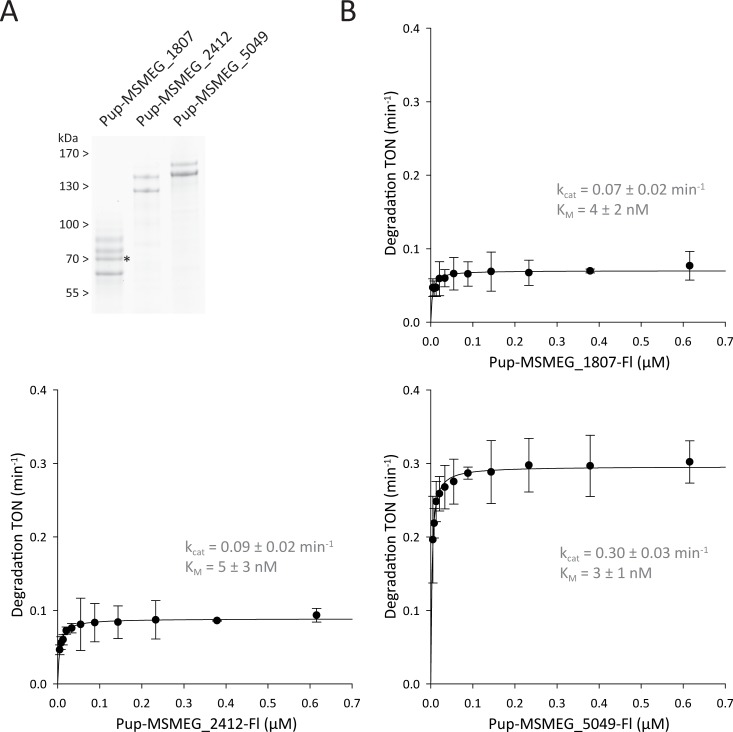
Michalis-Menten analysis of the Mpa-oCP dependent degradation of the three Pup-substrates identified in the pupylome degradation sample. (A) Purified, *in vitro* pupylated Msm substrates analyzed on Coomassie stained SDS-PA gel. The heterologously expressed target proteins were purified and subsequently pupylated *in vitro* at 3 μM concentration with 5 molar equivalents His_10_-3C-^Mtb^PupE and 1/3 molar equivalents ^Mtb^PafA followed by IMAC purification. The putative homodimers MSMEG_2412 and MSMEG_5049 are pupylated to 50%, likely reflecting pupylation of one protomer in each dimer. The dimeric MSMEG_1807 also exhibits pupylation of one protomer, but shows a more complex band pattern, since about half of the protomers of the unpupylated starting material were truncated. The full-length, unpupylated monomer signal is marked with an asterisk. (B) Fluorescence-based, real-time degradation assay of the three Pup-substrates identified in the pupylome degradation sample. Turnover numbers (min^-1^ per Mpa-CP) with a confidence interval of 2x standard deviation were plotted against the corresponding concentration of Pup-substrate-Fl and the curves were fitted to the Michaelis-Menten equation: TON = (k_cat_ * [S]) / (K_M_ + [S]).

The three distinct bands still visible on the Coomassie-stained gel after 24h of incubation were analyzed by tryptic digest and subsequent ESI-MS/MS analysis. The major components of the bands were determined to be MSMEG_5049 (136 kDa), 2-oxoglutarate dehydrogenase E1, MSMEG_2412 (121 kDa), a pyruvate carboxylase and MSMEG_1807/MSMEG_1813 (63/58 kDa), the α- and β-chains of a propionyl-CoA carboxylase. These four proteins were further analyzed after heterologous expression in *E*. *coli*. The β-chain of propionyl-CoA carboxylase was previously identified in a pupylome study as a false positive member of the pupylome, and in accordance with this report our attempts to pupylate MSMEG_1813 *in vitro* failed to produce pupylated product [27]. In fact, due to their biotin cofactor and their high abundance, carboxylases tend to be retained significantly in Strep-tag based affinity chromatography steps [39]. It is possible that a fraction then also carried over in the Ni^2+^-chelating affinity purification step. This would suggest that pyruvate carboxylase and propionyl-CoA carboxylase are both false positives. Nevertheless, we still tested the remaining three identified proteins in pupylation assays and showed that they could be pupylated *in vitro*, however not above 50% (Fig 6A). This is likely due to the fact that they form homodimers and pupylation of one subunit might sterically exclude pupylation of the second subunit, resulting in a dimer that is pupylated only on one protomer. The purified α-chain of propionyl-CoA carboxylase furthermore showed a more complex band pattern, since about half of the protomers of the unpupylated starting material were truncated at N- and C-terminus. Upon pupylation, all three proteins, despite their size and large number of potential target lysines, show one major pupylation band (in case of propionyl-CoA α-chain one each for the full length and the truncated versions). We used tryptic digest and ESI-MS/MS to determine the pupylated lysines, however only for MSMEG_5049 the sequence coverage was good enough to determine K837 as the major pupylated lysine.

We then fluorescently labeled the *in vitro* pupylated substrates to test them with the real-time degradation assay that we used for pupylated FabD, PanB, Icl1 and PckA (Fig 6B). While the k_cat_ values of degradation for the carboxylase proteins Pup-MSMEG_1807-Fl and Pup-MSMEG_2412-Fl are roughly half of the average rate for the previously tested substrates, Pup-MSMEG_5049-Fl is equally well degraded as Pup-PanB-Fl or Pup-FabD3KR-Fl. The observed K_M_ values do not exhibit significant differences to the ones measured for pupylated FabD, PanB, Icl1 and PckA (Table 2). Together, these results rule out the possibility that the substrates remaining after the 24 hour degradation time course display a particular intrinsic resistance to proteasomal degradation.

**Table 2:** Kinetic parameters for Mpa-oCP dependent degradation of the three Pup-substrates identified in the pupylome degradation sample.

	k_cat_ (min^-1^)	K_M_ (nM)	k_cat_/K_M_ (M^-1^ s^-1^)
Pup-MSMEG_1807-Fl	0.07 ± 0.02	4 ± 2	3.1 x 10^5^
Pup-MSMEG_2412-Fl	0.09 ± 0.02	5 ± 3	3.0 x 10^5^
Pup-MSMEG_5049-Fl	0.30 ± 0.03	3 ± 1	1.8 x 10^6^

The three substrates exhibit K_M_ values of degradation in the low nanomolar range similar to the substrates characterized in Fig 3. While the k_cat_ value of one of the substrates falls into the same range as the average value of the prior four substrates, two of the substrates range slightly below that value.

In summary our results show that all tested *in vitro* pupylated substrates are degraded with highly similar if not identical kinetic parameters, and the purified pupylome fades out as an ensemble during proteasomal degradation, suggesting that the Mpa-proteasome complex does not contribute differentiating specificity or selectivity towards different members of the pupylome.

### Dop exhibits differences in efficiency of depupylation for diverse substrates

**Fig 7:**
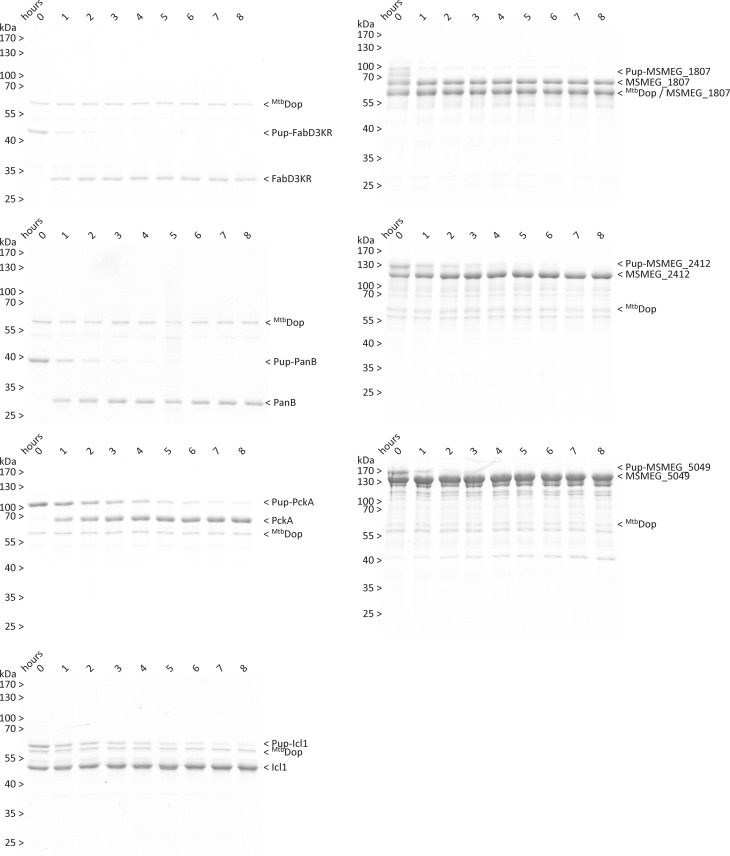
Depupylation time courses of Pup-substrate conjugates. Pupylated substrate (2 μM pupylated protomer) is completely turned over by 0.2 μM Dop in a time frame ranging from one hour to more than eight hours. Pup-FabD3KR takes little above 1 hour, while pupylated PanB, MSMEG_1807 and MSMEG_5049 are depupylated within 2 hours. MSMEG_2412 takes about 5 hours for the same amount of pupylated protomers, and PckA as well as Icl1 are not fully depupylated even within eight hours.

In light of the similarity of the kinetic parameters of degradation by Mpa-oCP for the substrates in this study, we compared their *in vitro* depupylation time courses at a 10-fold excess of pupylated protomer over depupylase Dop using SDS-PA gel-shift assays (Fig 7). Upon depupylation and the associated decrease in molecular weight, the bands for the pupylated substrates are shifted down to the unpupylated substrate band in SDS-PAGE. In contrast to what was observed for their proteasomal degradation, the pupylated substrates show a broad range of depupylation rates. Pupylated FabD3KR and MSMEG_1807 are fully depupylated by Dop within the first two hours of the recorded time course. MSMEG_5049, PanB and MSMEG_2412 are processed within about three to five hours, and PckA as well as Icl1 need eight or more hours to be fully turned over by the depupylase. This demonstrates that the interaction with and subsequent depupylation by Dop is more dependent on the individual substrate than Mpa-mediated binding and translocation into the proteasome. Likely, the interaction of Dop with Pup on a modified target more easily becomes subject to steric clashes at the interface of Dop and substrate, an interpretation also supported by the higher affinity of free Pup compared to Pup-substrate conjugate with Dop [37]. In particular, the placement of the isopeptide bond in the active site might be sterically challenging.

**Fig 8:**
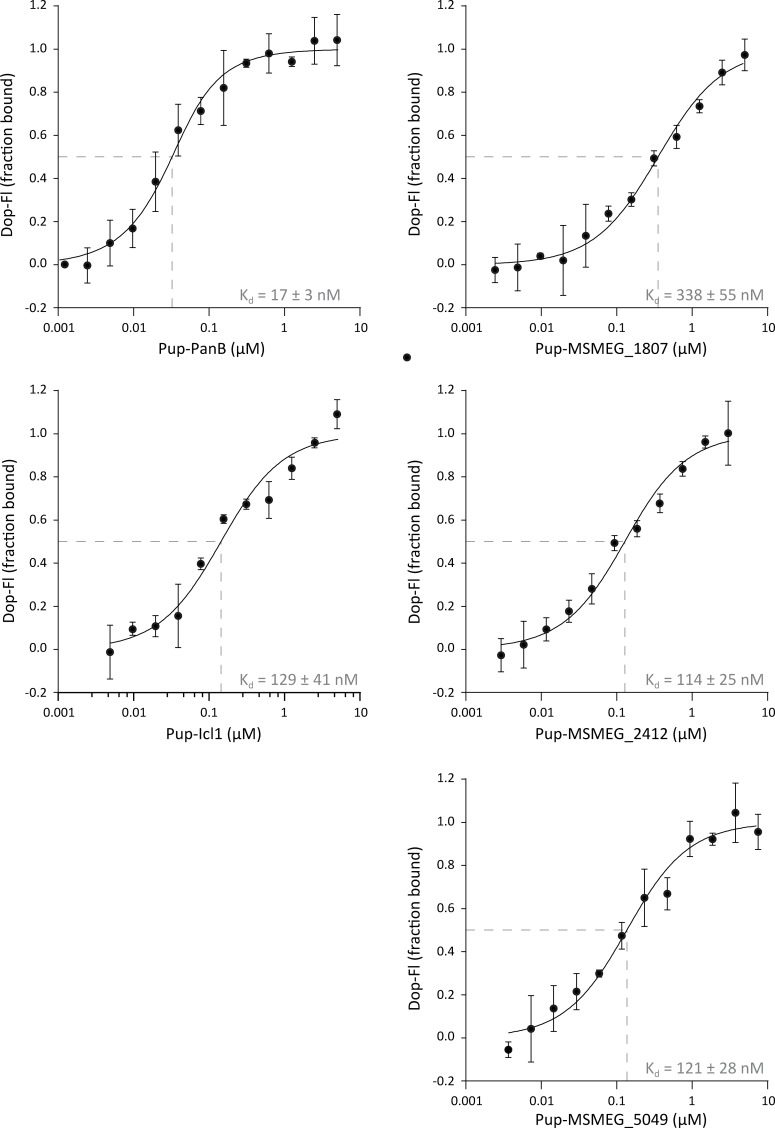
Binding of Pup-substrate conjugates to Dop in absence of nucleotide. Data was acquired with MST in a minimum of three replicates, by titrating pupylated substrate from 10 μM to 4 nM against fluorescently labeled Dop. The dependence of the bound fraction of fluorescein-labeled molecule (from normalized fluorescence signal change) on the concentrations of binding partner was fitted with the company-provided analysis software, yielding dissociation constants of 17 ± 3 nM for Pup-PanB, 129 ± 41 nM for Pup-Icl1, 339 ± 55 nM for Pup-MSMEG_1807, 114 ± 25 nM for Pup-MSMEG_2412 and 121 ± 28 nM for Pup-MSMEG_5049.

In addition to the gel-based depupylation assay, we measured the affinities of pupylated substrate to Dop in absence of nucleotide using MST. The dissociation constants were determined by titrating Pup-substrate to fluorescently labelled Dop (Fig 8). The determined K_d_s lie in the sub-micromolar range for all tested substrates in accordance with previously published results for Pup-FabD3KR [37]. With the exception of PanB-Pup that exhibits a K_d_ of 17 ± 3 nM, the other four tested substrates have a K_d_ in the high nanomolar range (129 ± 41 nM for Pup-Icl1 and 339 ± 55, 114 ±25 and 121 ± 28 nM for Pup-MSMEG_1807, Pup-MSMEG_2412 and Pup-MSMEG_5049, respectively). This result further supports the hypothesis that steric hindrance in positioning the isopeptide bond in the active site of Dop might be responsible for the observed differences in depupylation rather than differences in the overall affinity of pupylated substrate for Dop. The placement of the isopeptide bond in the active site is not expected to contribute significantly to the overall affinity, but its positioning is crucial for Dop activity.

## Discussion

In mycobacteria, the Pup-proteasome system has been shown to increase bacterial fitness under certain stress conditions, be it nitrogen starvation for *M*. *smegmatis* (Msm) or persistence in macrophages of *M*. *tuberculosis* (Mtb) [3–6]. Careful analysis of the structures and activities of the pupylation enzymes, namely the ligase PafA and the deamidase/depupylase Dop, has led to a good understanding of the reaction mechanisms by which pupylation and depupylation occur [9, 12, 13, 15–17, 40]. Furthermore, several studies have been conducted to identify pupylated substrates of different actinobacterial organisms, generating a catalog of pupylation targets [8, 27–34]. However, few key examples of Pup-modified proteins have been found, that upon degradation can increase the chance of survival under certain conditions [41, 42]. While for the substrates in question and a few others degradation has been shown, it is still unclear why many members of the pupylome show no difference in steady-state levels with impaired proteasomal degradation. For this reason, we investigated the possible discrimination between different pupylated proteins as degradation targets for the Mpa-proteasome complex.

Before the pupylated substrate is engaged into the translocation pore of Mpa, it must be recognized by Mpa. This initial recognition step is best reflected in the affinity of the Mpa-ring to the individual substrate proteins in absence of nucleotide, where ATPase driven pore engagement cannot take place. The recombinantly produced, bona fide pupylated substrates tested in this study displayed within error the same dissociation constants to Mpa, while Pup in the absence of substrate binds with a K_d_ roughly one order of magnitude higher. This finding suggests on the one hand additional contacts between the substrate portion and Mpa, aside from the shared, three-stranded coiled-coil formed with the Pup portion. On the other hand, given the diverse nature of the tested substrates and the fact they nevertheless show very similar affinities, these additional contacts likely comprise a collection of weak unspecific interactions. As we know from our previous analysis that Pup occupies only one of the three coiled-coils, a possible scenario is that the two unengaged coiled-coils interact with the substrate portion of the conjugate [19, 43]. In the broader context of the cell, this means that pupylated substrate is preferentially bound by Mpa compared to Pup alone, ensuring both efficient degradative turnover and the direction of free Pup to the ligase PafA for modification of substrate.

We then tested the affinity of pupylated substrates under threading conditions by determining K_M_ values for the degradation reaction, since differences in the efficiency of engaging the substrate into the pore should be expressed in that parameter. While the dissociation constants in absence of nucleotide for different pupylated substrates to Mpa lie around 0.2 μM, the K_M_ values of degradation for these same substrate proteins are almost two orders of magnitude smaller. This means that upon ATP-dependent pore engagement and subsequent threading into the degradation complex, substrates have little chance of escaping back out of the pore conduit, resulting in a greatly reduced off-rate once the substrate is engaged. This ensures that no degradation intermediates can escape and accumulate in the cell. Analogous to the Michaelis constants falling into a narrow range for different substrates, also the catalytic rates of degradation for these proteins show little difference amongst one another. Evidently, all the tested substrates are turned over by the degradation machinery with similar rates. The minor differences in the observed k_cat_ values for the recombinantly produced Mtb substrates could potentially be attributed to a mild avidity effect of Pup-modifications on each of the protomers in the PanB decamer. Nevertheless, the differences are too small to serve a discriminatory function between different members of the pupylome.

The Msm substrates identified from the degradation of purified pupylome as potentially slower degradation candidates displayed 2–3 fold reduced k_cat_ values for two of the substrates, and the third substrate showed the same degradation rate as pupylated PanB. Particularly in the case of the two carboxylase proteins (MSMEG_1807 and MSMEG_2412), it is likely that they are false positives carried over due to their biotin cofactors. Furthermore, the fact that the signal for MSMEG_5049 and MSMEG_2412 in the anti-Pup blot is weak insinuates that they might be present for reasons other than slow degradation and may not even be significantly pupylated. Both MSMEG_2412 and MSMEG_5049 harbor a large number of histidines, some of which are located closely together within the sequence, increasing the chance for unspecific binding to the metal chelating resin under denaturing conditions. MSMEG_1807, the only protein with a clear signal remaining on the anti-Pup Western blot after 24 hours, shows with 0.07 min^-1^ the slowest k_cat_ value. It is possible that this mildly reduced turnover together with an overall high abundance could lead to MSMEG_1807 being left over. MSMEG_2412 and MSMEG_5049 are likely present in low micromolar concentrations, and MSMEG_1807 may even be present at concentrations of up to 15 μM [44]. The pupylome will contain larger amounts of very abundant pupylatable substrates, particularly if they exist as oligomers and unpupylated protomers might copurify. This also reveals a flaw in the preparation and analysis of all pupylomes that were published so far: there is a certain bias towards larger and especially more abundant proteins in the cell, as these will be more easily detected with both gel-based and mass spectrometric analysis methods. Low abundance pupylation targets might therefore fall below the detection limit of many methods, just like the “lonely guy” identified instead in a genetic screen [41].

A rather different picture emerges for the activity that opposes degradation of the pupylated substrates by removing the Pup-modification. While previous data has shown that the deamination of Pup by Dop to render the tag ligation-competent is happening very fast, depupylase Dop processes different pupylated substrates with varying efficiency, so that removal of Pup could become a discerning factor in determining the fate of a pupylated protein. Particularly under conditions where the proteasome works at full capacity on a diverse range of substrates, the fast depupylation of certain pupylome members might ultimately prevent their degradation. This could be a mechanism to ensure that certain substrates are pupylated only transiently or that substrates that should not be pupylated in the first place are removed from this pool quickly.

The ratio of substrate to enzyme between the degradation reaction and the depupylation reaction differ between assays, to ensure good visibility in the gel readout for either time-course. As a result, the observation time frame for the pupylome degradation had to be longer than the one for depupylation. However, we assume that the small difference between degradation speed of substrates, together with the larger difference observed in depupylation speed between substrates are sufficient to lead targets preferably to one or the other pathway, particularly under conditions where substrates are present simultaneously and competing.

In our work, we have been able to show that *in vitro* pupylated substrates are degraded with similar catalytic parameters by the Mpa-oCP complex, and substrate specificity is likely not achieved at the level of the ATPase. However, to date it is still unknown how in the cell the proteasome is activated to a fully functional degradation complex with Mpa. Also, the interplay of the Pup ligation catalyzed by PafA and the depupylation activity of Dop on the dynamics in the pupylome, as well as regulation of the PPS enzyme levels are unclear, although it has been shown that the pattern of pupylation changes during different phases of growth in mycobacteria [5]. As proteasomal degradation itself appears to lack specificity, one might expect that the balance between pupylation and depupylation is responsible for directing substrates towards degradation or recovery, indicated also by the larger range in depupylation efficiency displayed by Dop for different pupylated substrates. Our experiments were performed *in vitro*, using isolated one-enzyme (complex) reactions with purified components. It is possible that in the cellular environment, the interplay between the components of the PPS is influenced by additional factors. Further experimentation is therefore needed to study substrate flow within the PPS *in vivo*, with particular attention given to finding potential cellular factors that regulate the components of the PPS.

## Materials and methods

Chemicals were purchased from Sigma-Aldrich and Media from Difco, if not otherwise stated.

### Cloning, expression and purification

Wild-type ^Mtb^Mpa (Mpa), open-gate ^Mtb^proteasome complex (oCP), ^Mtb^PupE variants and ^Mtb^PafA-His_6_ were expressed and purified as described [10, 25]. Degradation substrates were cloned according to the FX-cloning protocol into pBXNH3 vectors and transformed into *E*. *coli* MC1061 cells for expression [45]. Expression cultures were shaken in Terrific Broth (TB) medium supplemented with 120 μg/ml Ampicillin at 37°C to an OD_600_ of 1.2 before temperature reduction to 25°C for 1 hour and subsequent induction of overnight protein expression by addition of 0.008% L-Arabinose (w/v). Cells were harvested by centrifugation at 6000 rpm, 4°C for 10 min (Sorvall RC 61, F9S rotor), diluted 1:2 (w/v) in phosphate-buffered saline (PBS: 15 mM Na_2_HPO_4_, 1.8 mM KH_2_PO_4_ (-HCl to pH 7.4 @ 25°C), 137 mM NaCl, 2.7 mM KCl) with the addition of Complete EDTA-free protease inhibitor (Roche) and disrupted by three passes through a M-110L microfluidizer (Microfluidics^TM^) at 11000 psi maximal chamber pressure, before centrifugation at 45000 rpm, 4°C for one hour (Beckmann L90-K, 70 Ti rotor). The His_10_-tagged, overexpressed proteins were purified by immobilized metal affinity chromatography (IMAC) by passing the supernatant through a 5 ml HiTrap HP column (GE Healthcare) charged with Ni^2+^. The column was washed with 10 column volumes (CV) wash buffer (50 mM HEPES-NaOH (pH 7.5 @ 4°C), 500 mM NaCl, 10% glycerol, 50 mM Imidazole) to remove non-His-tagged proteins before elution of tagged proteins in 5 CV elution buffer (50 mM HEPES-NaOH (pH 7.5@4°C), 150 mM NaCl, 10% glycerol, 300 mM Imidazole). After addition of 1:50 molar equivalents (m_eq_) of Human Rhinovirus (HRV) 3C protease to cleave off His tags, the eluate was dialyzed against 500 ml reaction buffer (rxnB(7.5): 50 mM HEPES-NaOH (pH 7.5@25°C), 150 mM NaCl, 10% glycerol) at 4°C overnight. The dialyzed sample was subjected to reverse IMAC, collecting the flowthrough with 2 CV addition of wash buffer before concentrating the sample to ≤5 ml with Amicon Ultra centrifugal filters (Millipore) and loading on a Superdex 200 (20/26) pg (GE Healthcare) column connected to an Äkta purifier system at 4°C for final purification and buffer exchange into rxnB(7.5). Protein fractions from the gel filtration were pooled and concentrated again before determining concentrations with a Nanodrop ND-1000 spectrophotometer (Fischer) and flash-freezing the aliquoted protein in liquid N_2_ for storage at -20°C.

### Preparative pupylation and subsequent purification of pupylated targets

5 μM substrate protein were incubated with 20 μM His_10_-3C-^Mtb^PupE and 1 μM ^Mtb^PafA-His_6_ in rxnB(8.0) (like rxnB(7.5) but titrated to pH 8.0 at 25°C), supplemented with 20 mM MgCl_2_ and 5 mM ATP in 500 μl aliquots to a final volume of 5 ml at 25°C for 20 h. Afterwards, the reaction mixture was passed through a 1 ml HiTrap HP column (GE Healthcare) charged with Ni^2+^ and equilibrated with rxnB(8.0). The column was washed with 10 CV wash buffer before elution of pupylated proteins along with PafA-His_6_ in 10 CV elution buffer. The elution fraction was supplemented with 1:5 m_eq_ of HRV 3C protease and dialyzed against 200 ml of rxnB(7.5) at 4°C for 3 hours. The sample was then subjected to reverse IMAC, with collection of flow through (FT) and 5 CV wash buffer. The FT/wash pool was concentrated to ≤ 500 μl on Amicon Ultra centrifugal filters (Millipore) and subjected to gel filtration on a Superdex 200 (10/300) GL column (GE Healthcare) connected to an Äkta Purifier system at 25°C in rxnB(7.5). Fractions containing pupylated protein were pooled and concentrated to 20–100 μM (protomer).

### Fluorescence labeling of pupylated substrate and Mpa

Purified Pup-substrates were labeled with 5-/6-carboxyfluorescein succinimidyl ester (NHS-Fluorescein, Fischer) targeting terminal amino groups on the protein surface. Typically, 20–100 μM of substrate (protomer) in ≤ 500 μl rxnB(7.5) were incubated with 2–5 m_eq_ of NHS-Fluorescein (10 mg/ml in DMSO) for 2 hours at 25°C. The reaction mixture was then subjected to gel filtration on a Superdex 200 (10/300) GL column (GE Healthcare) connected to an Äkta Purifier system at 25°C in rxnB(7.5) to remove excess label. Fractions containing protein were combined and concentrated on Amicon Ultra centrifugal filters (Millipore). Concentrations and degree of labeling were calculated by measuring absorbance at 280 and 493 nm on a NanoDrop ND-1000 spectrophotometer (Fischer), assuming a molar extinction coefficient of 70’000 M^-1^cm^-1^ for Fluorescein and a correction factor of 0.167 for the A280/A493 ratio of Fluorescein. Yields were typically around 90% of initial protein recovered with labeling efficiencies between 0.8–4 labels per protomer, termed Pup-Substrate-Fl below.

Wild-type ^Mtb^Mpa was labeled with Fluorescein-5-Maleimide (Fischer) in cystein-specific linkage. 25 m_eq_ of the Fluorophore (10 mM in DMF) were added to 8 μM of wild-type ^Mtb^Mpa in rxnB(7.5) supplemented with 10 mM EDTA in a reaction volume ≤ 500 μl and incubated for 2 hours at 25°C. The reaction mixture was purified as described above and concentrations were calculated by measuring absorbance at 280 and 495 nm on a NanoDrop ND-1000 spectrophotometer (Fischer) with sample diluted 1:2 into 100 mM BisTrisPropane (pH 9.0 @ 25°C), 150 mM NaCl, 10% glycerol, assuming a molar extinction coefficient of 70’000 M^-1^cm^-1^ for Fluorescein and a correction factor of 0.121 for the A280/A495 ratio of Fluorescein. Yields were 95% initial protein recovered with a labeling efficiency of 2.96 labels per Mpa protomer, in good agreement with the three cysteine residues on the protein. Fluorescently labeled Mpa is termed Mpa-Fl below.

### Microscale thermophoresis (MST) measurements

Dissociation constants between Mpa_6_ and oCP, Mpa_6_ and Pup/Pup-Substrate or Dop and Pup-Substrate were determined by MST measurements on a Monolith NT.115 device (Nanotemper) in 3–8 replicates. Typically, 5 nM of Mpa-Fl, 40–150 nM of Pup-Substrate-Fl or 30 nM Dop-Fl were incubated with a serial dilution of binding partner in concentrations ranging from 10x over to 10x below estimated K_d_ in rxnB(7.5) supplemented with 1 mM MgCl_2_ and 0.05% Tween-20 in 10 μl reaction volume. Measurements were performed in MST Premium Coated Capillaries at 17°C start temperature with 20% LED power and 20–80% MST power, corresponding to a temperature jump of 2–8°C, depending on the target protein. The measurement sequences were configured as follows: 5 s base-lining, 25 s IR laser on, 5 s off, 30 s recovery before next measurement. Data was analyzed with the company-provided MST Affinity Analysis software v2.0.2 using the embedded Thermophoresis and T-jump analysis module and the attached K_d_ calculation model to fit the data [38].

### Degradation assay with fluorescently labeled substrates

Fluorescently labeled Pup-substrates (labeling procedure see above) were subjected to degradation by Mpa-oCP. Degradation time courses were followed by measuring the fluorescence increase due to unquenching of the fluorophore upon protein breakdown.

With the exception of Pup-PanB-Fl Pup-Substrate-Fl and Mpa were kept at a constant 10:1 ratio of pupylated protomer to Mpa hexamer for all velocity measurements. Pup-PanB-Fl was kept at a constant ratio of pupylated protomer to Mpa hexamer of 100:1. Mpa and Pup-Substrate-Fl in rxnB(7.5) were serially diluted corresponding to concentrations ranging between 615 and 4 nM pupylated protomer and added into 96 well black polystyrene half area plates with non-binding surface (Corning). Additionally, the reaction mixture contained 0.01% Tween-20, 20 mM MgCl_2_, 2 μM oCP and 0.01 U/μl creatine phosphokinase. Reactions were started by addition of 10 mM ATP and 10 mM phosphocreatine in 50 μl final reaction volume. Fluorescence traces were recorded in a Synergy 2 plate reader (BioTek) with λ_ex_ at 485/20 nm and λ_em_ at 525/20 nm and a 510 nm top filter at 25°C in 40 s intervals, including 3 s of shaking on medium setting between measurements. Three independent replicates were measured and after baseline subtraction, the linear phase of the reaction between 400–1400 s, corresponding to ≤10% of substrate degradation was fitted to extract the slopes and calculate turnover numbers (TONs) per Mpa_6_-oCP. They were then plotted against substrate concentration with a confidence interval of 2x standard deviation, before fitting to the following equation: TON = (k_cat_ * [S])/(K_M_ + [S]) using SigmaPlot 12 (Systat Software) to determine the catalytic parameters of degradation.

### *M*. *smegmatis* pupylome: Production–degradation–sample preparation

Preparation of glycerol stocks: 200 ng of plasmid DNA (Strep-TEV-His6-MtbPupE_pMyC) were incubated 10 min with 100 μl competent *M*. *smegmatis* (Msm) SMR5*Δdop* cells [16] on ice before electroporation in a 0.2 cm GenePulser cuvette (BioRad) at 2.5 kV for 5 ms. 700 μl of 7H9 medium supplemented with 0.02% Tween-80 were added and cells were recovered by shaking 4 hours at 37°C. 200 μl of cell suspension were spread on LB/Agar supplemented with 50 μg/ml Hygromycin B and 100 μg/ml Streptomycin (Carl Roth) and incubated for 3 days at 37°C until colonies appeared. A single colony was picked and grown at 37°C in 7H9 medium, supplemented with 50 μg/ml Hygromycin B, 100 μg/ml Streptomycin and 0.02% Tween-80. 20% glycerol stocks were prepared and flash frozen in liquid N_2_ before storing them at -80°C.

Expression of affinity-tagged pupylome in Msm: 100 ml 7H9 supplemented with 50 μg/ml Hygromycin B, 100 μg/ml Streptomycin and 0.02% Tween-80 were inoculated with a Msm glycerol stock and grown at 37°C for two days. The preculture was diluted 1/200 into the main culture (7H9 medium supplemented with 50 μg/ml Hygromycin B, 100 μg/ml Streptomycin, 0.02% Tween-80 and 2 g/l D-Glucose, 1 l medium per 5 l unbaffled Erlenmeyer flask). Main cultures were shaken at 150 rpm for 24 h at 37°C to an OD_600_ of 0.8–1 before induction with 0.2% Acetamide (final, sterile filtered 20% stock in H_2_O) and further incubation for 10 h at 37°C, 150 rpm. Cells were harvested by centrifugation at 7000 rpm, 15 min, 4°C (Sorvall RC 6+, F9S rotor) and cell pellets were flash frozen in liquid N_2_ and stored at -20°C until further use.

Purification: Msm pellets were diluted 1:2 (w/v) into 50 mM HEPES-NaOH (pH 7.4 @ 4°C), 150 mM NaCl with the addition of Complete EDTA-free protease inhibitor (Roche). The suspension was homogenized on ice at 10’000 rpm (Heidolph Diax900 ultra turrax) before cell emulsification for 10 passes at 11000 psi maximum chamber pressure on a M-110L microfluidizer (Hyland Scietific). The resulting suspension was centrifuged 2x5 min at 3’000 g (Eppendorf 5804R) and 2 hours at 45’000 rpm, 4°C (Beckmann L90-K, 70 Ti rotor) to remove cell debris. The supernatant was loaded on 2x5 ml StrepTrap HP columns (GE Healthcare) at 3 ml/min before washing 20 CV with rxnB(7.5). Pupylated proteins were eluted in 8 CV of the same buffer containing 2.5 mM Desthiobiotin. Solid Ammonium sulfate (AS) was added to 90% saturation to the solution and stirred mildly at 4°C until dissolved. Precipitated protein was collected by centrifugation at 20’000 rpm, 4°C for 15 min (Sorvall RC 6+, SS-34 rotor). The AS precipitated protein pellet was dissolved in 1 ml dialysis buffer (rxnB(7.5) supplemented with 2 mM EDTA, 5 mM β-Mercaptoethanol) before addition of 3 μM Tobacco Etch Virus (TEV) protease (final) and dialysis versus 1 l of rxnB(7.5) for 4 hours and 2 l of fresh buffer overnight at 4°C. The dialyzed protein solution was centrifuged at 15’000 rpm, 4°C for 15 min (Sigma 2K15) and transferred to a fresh tube before measuring final concentration. Yields were typically about 3% of the total protein mass in the supernatant estimated from Bradford assay.

Pupylome degradation *in vitro*: For the degradation assays, 0.3 mg/ml of purified pupylome were supplemented with 0.2 μM Mpa_6_, 0.2 μM oCP, 20 mM ATP, 100 mM Phosphocreatine, 0.01 U/ml creatine phosphokinase, 20 mM MgCl_2_ and incubated at 37°C in 500 μl aliquots. At the indicated time points, the degradation reaction was quenched by addition of 350 mg solid Guanidinium chloride (GdmCl, NIGU) and 1 ml of denaturing buffer (50 mM HEPES-NaOH (pH 7.4 @ 4°C), 150 mM NaCl, 6 M GdmCl) and incubation at room temperature for at least 4 hours. The samples were purified over a 1 ml HiTrap HP column (GE Healthcare) charged with Co^2+^. The flowthrough (FT) was combined with the wash fraction (W, 50 mM HEPES-NaOH (pH 7.4 @ 4°C), 500 mM NaCl, 6 M Gua) to a total volume of 20 ml. His-tagged proteins were eluted in 10 CV (E, 50 mM HEPES-NaOH (pH 7.4 @ 4°C), 150 mM NaCl, 6 M GdmCl, 1 M imidazole). The FT/W and E fractions were dialyzed against H_2_O to reduce the concentration of GdmCl to roughly 250 mM. The dialyzed samples were transferred to SS-34 centrifugation tubes, and volumes were adjusted to 30 ml with H_2_O. Sodium desoxycholate was added to 0.1% (10% stock) followed by vortexing before the addition of 10% trichloroacetic acid (100% stock, w/v) and subsequent vortexing. The samples were incubated at 4°C on a rolling shaker for 20 min, then centrifuged at 20’000 rpm, 4°C for 20 min (Sorvall RC 6+, SS-34 rotor). The supernatant was discarded by decanting and pipetting, then the pellet was dissolved in 1.5 ml ice-cold acetone. After thorough vortexing, the solutions were transferred into 2 ml Eppendorf tubes and incubated at -20°C for 45 min before centrifugation at 15’300 rpm, 4°C for 20 min (Sigma 2K15). The supernatant was removed by pipetting, and the remaining pellet was dissolved in 2 ml ice-cold Ethanol, vortexed thoroughly and incubated at -20°C for 1.5 hours before centrifugation at 15’300 rpm, 4°C for 20 min (Sigma 2K15). The supernatant was removed by pipetting and the pellets were dried overnight at room temperature. The remaining pellet was dissolved in 30 μl 1x reducing SDS loading dye. The samples were analyzed by SDS-PAGE and Western Blotting using polyclonal α-Pup-(rabbit) antibodies and HRP-linked α-rabbit-(mouse) antibodies.
